# The Pathology and Treatment of Pulmonary Tuberculosis; and on the Local Medication of Pharyngeal and Laryngeal Diseases Frequently Mistaken for, or Associated with, Phthisis

**Published:** 1854-04

**Authors:** 


ART. VIII.— *The Pathology and Treatment of Pulmonary Tuberculosis; and on the local medication of Pharyngeal and Laryngeal Diseases frequently mistaken for, or associated with, Phthisis.* By JOHN HUGHES BENNETT, M. D., F. R. S. E., Professor of the Institutes of Medicine and of Clinical Medicine in the University of Edinburgh, etc., etc., etc. Edinburgh: Sutherland & Knox. 1853.

We are indebted to the author for an early copy of this very valuable monograph. Dr. Bennett has distinguished himself by his early advocacy of the nutritive treatment of tuberculosis. We trust that this volume may be reproduced in this country, as it contains, in a concise and well-arranged form, the present knowledge of tubercle. As it is not probable that our readers will soon have an opportunity of purchasing the book, we have chosen to give rather an abstract of its contents, than a review of its doctrines. While the course we adopt involves much more labor than a mere critical notice, we shall be repaid, if, by our tabulation, we present to our readers, in a condensed shape, the modern discoveries and doctrines of tuberculosis.

The first chapter is devoted to the pathology of pulmonary tuberculosis.

*Early Symptoms.* Bad and capricious appetite, furred or morbidly clean tongue, unusual acidity of the stomach and alimentary canal, anorexia, constipation alternating with diarrhœa, and a variety of symptoms denominated dyspeptic.

*Histology of Tubercle.* Miliary, infiltrated, or encysted. Structure in all these the same; the differences depending upon the extent and age of the exudation.

*Physical appearances.* Yellowish or dirty white color, consistence varying from that of tough cheese to that of cream. On section when tough it is smooth and waxy; when soft, slightly granular.

*Microscopy.* Tubercle corpuscles are irregular shaped bodies, round, oval, or triangular; longest diameters varying from $\frac{1}{4000}$ to $\frac{1}{2000}$ of an inch. They are composed of a distinct wall containing three or more granules, without a nucleus, and mixed with numerous granules and molecules, from a size scarcely measurable to a diameter of $\frac{1}{6000}$ of an inch. Acetic acid renders the corpuscles more clear, and dissolves many of the granules. Ether and alcohol produce no change. Ammonia partially, and potash completely, dissolves the corpuscles. The gray semi-transparent granulation, though harder and different to the eye, is the same microscopically, only more transparent and less defined.

The calcareous tubercle presents fragments of phosphate of lime and crystals of cholesterine. On old tubercle we frequently find pigment cells forming a zone around the mass.

### *Distinctions between Tubercle and other Corpuscles.*

| *Tubercle.* | *Pus Corpuscle.* |
|---|---|
| Acetic acid causes the granular nucleus to appear. | No granular nucleus or addition of acetic acid. |
| | *Plastic Corpuscles.* |
| Irregular form, small size, absence of primitive filaments. | Regular rounded form, large size, and presence of primitive filaments. |
| | *Granular Corpuscles.* |
| Small size, yellowish color, no nuclei. | Large size, brownish or blackish color, nucleated or granular. |
| | *Cancer Cell.* |
| Small size, opaque, non-nucleated. | Large, transparent, and distinctly nucleated. |
| | *Reticulum of Cancer.* |
| | Closely resembles tubercle, but is always accompanied by cancer cells. |

*Chemical Composition.*—Tubercle consists of animal matter mixed with earthy salts, of which the relative proportion varies. There is most animal matter in recent, most earthy matter in chronic tubercle.

Animal matter contains:

Albumen; a large amount.
Caseine; probably.
Gelatine; doubtful.
Phymatine; doubtful.
Fibrin; in small but variable proportion.
Fat; in small but variable proportion.

The earthy salts are composed of

Insoluble phosphate of lime.

Insoluble carbonate of lime.

Soluble salts of soda.

The ultimate constituents of tubercle are the same as those of other compounds of protein.

*Nature of Tubercle.*

| | | |
|---|---|---|
| *Inflammatory exudation* occurs at all epochs of life, in all tissues, in large or small quantity, with greater or less rapidity. Is attended by inflammatory symptoms, with a tendency to cell formations, which rapidly break down and are absorbed or excreted.—When chronic, tends to fibrinous formations, producing adhesions, etc. | *Cancerous exudation* occurs in adult or advanced life, in every tissue, most common in glandular or fatty organs, as the liver, or mamma; occurs in lymphatic glands *secondarily;* slow in progress when fibrous; rapid when corpuscules abound; tends to perfect formations of cell life, which self-develop and spread, and ulcerate in exuberant fungoid excrescences. | *Tubercular exudation* occurs in young subjects, occurs in all tissues, but most common *primarily* in lymphatic glands, or fibrous or albuminous textures; progress slow, very imperfect cell formation, little tendency to absorption, but great disposition to disintegration and ulceration; and is preceded by derangements of the primæ viæ. |

Of the three kinds of exudation, tubercle is the lowest, pus the medium, and cancer the highest in the scale.

*Blood changes.* The constitution of the blood determines that of the exudation.

In simple exudation the blood is normal.

In cancerous it contains an excess of nutritive material.

In tubercle it is deficient in nutritive material.

Tubercle is a coagulated exudation, occupying the same seat as other exudations. It transudes in a fluid state through the capillaries to some extent into the cellular portion of the lung, but mostly into the air vesicles, where there is least vascular resistance.

Why does this exudation occur in phthisis? The philosophy of healthy nutrition consists in the ingestion of proper organic food, its digestion into albuminous and fatty compounds, their absorption into the lacteals, and their transformation into chyle corpuscles, by the inclosure of an oil globule in a cell wall, formed from the albuminous blastema. To this absorption an emulsion is necessary; the alkaline secretions of the pancreas make that emulsion; and, finally, in phthisis this pancreatic juice is neutralized by the acidity of the gastric and intestinal secretions, so that a proper emulsion is not formed, the absorption of the albumen is hastened and favored thereby, while that of the fat is retarded or prevented. There is, then, an undue proportion of albumen in the blood; the fats of the tissues are absorbed to supply the deficiency of that element; the frame is emaciated; the lungs become liable to local congestions; albuminous exudations take place, which are tubercles.

The objection that fat is found in the blood of phthisical patients is explained by its absorption from the tissues. That fat is also found in tubercle itself, Dr. Bennett ascribes to a process of degeneration, like that of the conversion of muscular tissue into adipocere.

*Natural process of Tubercle.* Tubercle deposited in the parenchyma of the lung, and thence passing into and filling up the air vesicles, is a foreign body. It disintegrates, dies; its substance softens and is expectorated; with it is destroyed that portion of the lung which it occupied, but no more; it has no tendency to spread its ravages. Supposing the process of deposit to be arrested here, the sides of the cavity approximate and unite, forming a cicatrix; but oftener the deposit is continued: other tubercles soften and are excreted: ulcerations exist side by side: the vitality of the tissue intermediate is destroyed: it, too, perishes: large cavities are formed thus from numerous smaller ones.

The life of tubercle is various. It, like every other tissue, (a fact beautifully explained by Paget) has its allotted period of existence. Its organization is low; its vitality is brief; if the condition of the primæ viæ becomes healthy, it may either ulcerate and cicatrize, or become inclosed in a fibrinous cyst, its albumen and fat be absorbed, and the earthy portions remain. This is the more common course. The presence of calcareous concretion is an evidence of long life in the tubercle, and of partial natural cure.

Following this history of tubercle, of which we have endeavored to present the prominent features, Dr. Bennett gives cases of recovery from phthisis in advanced stages, which were verified by post-mortem examinations when the patient had died from other causes.

As will have been noticed, but one conclusion can be drawn from this view of tubercle. It is the result of imperfect digestion and assimilation.

Hence it occurs at that age when the system is building up, and nutrition is most active. Able-bodied men subjected to privation are not especiably liable to it for this reason, while those engaged in sedentary employments, and subjected to the causes of dyspepsia, are most apt to contract it. Cases are cited illustrating the influence of poor diet, want of light, air, and ventilation in the causation of this disease.

*The Diagnosis.* Consumption, says Dr. Bennett, is not especially an incurable disease, except as it is insidious in its advent. An early diagnosis is most important. The development, elimination, and mode of arrest of tubercle, all indicate that were it early detected, and the proper attention directed to the digestive functions, the deposit might often be checked, and that already deposited removed, either by expectoration or absorption; thus leaving the lung but little impaired. But it is in diagnosis where medical men are deficient. Though physical exploration is much talked of, it is too true that few practitioners are capable of ascertaining the early and finer differences of sound.

We may be pardoned if we here digress from the volume before us, and allude to the fact that physical diagnosis has no true standard of departure. We take some portion of the lung of each case as an ideal by which we judge the remainder; but how little do we know of those normal variations in pitch, resonance, and intensity, which are within the limits of health. It has already been demonstrated by Dr. Flint, that the pitch of bronchial respiration is subject to certain conditions, which are variations as we compare side with side, but which are uniform and healthy as we compare individual with individual. A careful analysis of some hundreds of healthy chests would furnish a positive standard as to normal differences in different localities of the lung, which is much needed. Such an analysis we have reason to believe will ere long be presented to the profession.

Our space will not permit a lengthy *resume* of the treatment approved by Dr. Bennett. It will, however, suggest itself from the pathology of the disease, and we give his indications:

"*First Indication. To improve the Faulty Nutrition, which is the Cause of the Exudation assuming a Tubercular Character.*"

In many cases we find the tone of the stomach so impaired as to reject such fatty animal food as is called for by this indication. Hence it is often necessary to afford the fat in a fluid form, which may easily be absorbed and assimilated. He regards cod-liver oil simply as an analeptic. It is readily digestible; it combines easily with albumen to make the chyle corpuscle; it nourishes the body; checks exudation, and diminishes cough.

"*Second Indication. To favor Absorption of the Exudation already poured out, and subdue the Symptomatic Fever produced.*"

The problem in the treatment of tubercle is, how to meet the opposing indications of invigorating the general system, while we combat the local and intercurrent inflammatory action. Dr. Bennett considers the propriety of bleeding, but objects to it. The plan he adopts is similar to that which guides a typhus fever. For many years he has relied upon small doses of antimony and diuretics to combat the acute symptoms. In chronic forms he would counter-irritate.

"*Third Indication. To prevent the Recurrence of fresh Exudations by careful attendance to Hygienic regulations.*"

Under this indication he considers the influences of climate, diet, and other hygienic influences.

Our limits are filled. We commend the book to those who can procure it, as the latest, the most concise, able, and positive information to be obtained upon the subject of pulmonary tuberculosis. H.

